# Supplementary material for: The H2B deubiquitinase Usp22 promotes antibody class switch recombination by facilitating non-homologous end joining
Source: Nat Commun. 2018 Mar 8;9:1006. doi: 10.1038/s41467-018-03455-x (PMC5843634; doi:10.1038/s41467-018-03455-x)
Supplement: Supplementary file 1 — Supplementary Information [file 41467_2018_3455_MOESM1_ESM.pdf]

The H2B deubiquitinase Usp22 promotes antibody class switch recombination by facilitating non-homologous end joining

Li et al

Supplementary Information

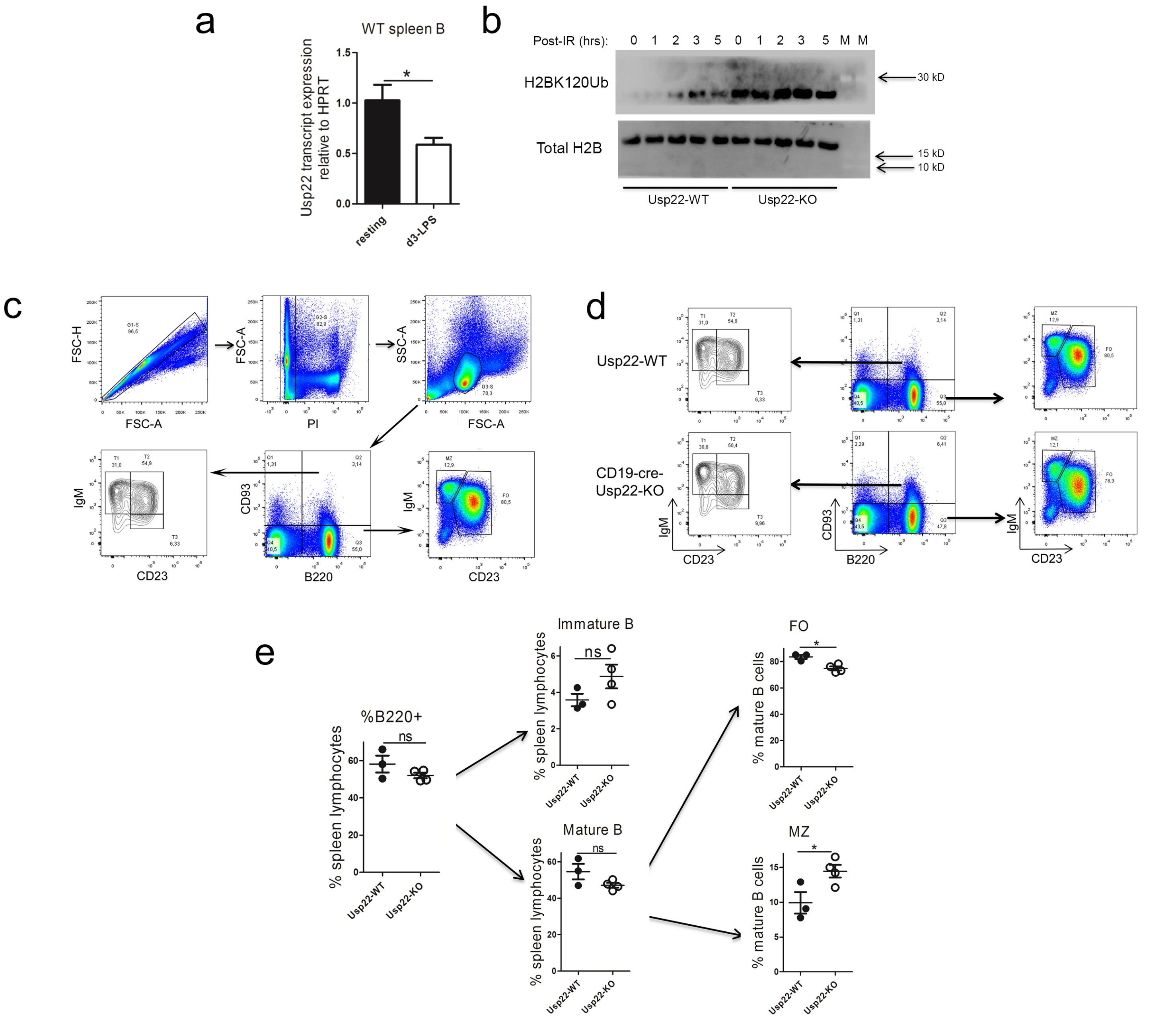

Supplementary Figure 1. B cell profile analysis in the spleen of CD19-cre-Usp22 KO and WT littermates. (a) qPCR analysis of Usp22 mRNA was performed with resting or d3-LPS stimulated WT spleen B cells (n=3 mice per group). Data represent two independent experiments. (b) Uncropped western blot images for Figure 1d (M: marker). (c) Gating strategy of the B cell profiling in the spleen. (d) Representative FACS plots of spleen B cells from CD19-cre-Usp22 KO and WT littermates. (e) The relative percentage analysis of B cells of indicated subsets in spleen. There was no major alterations of B cell profiles in the spleen of CD19-cre-Usp22 KO mice, compared to WT littermates. Data in (c-e) represent two independent experiments each with three to four mice per group (ns: not significant); data in (a and e) were analyzed using two-tailed unpaired Student t test.

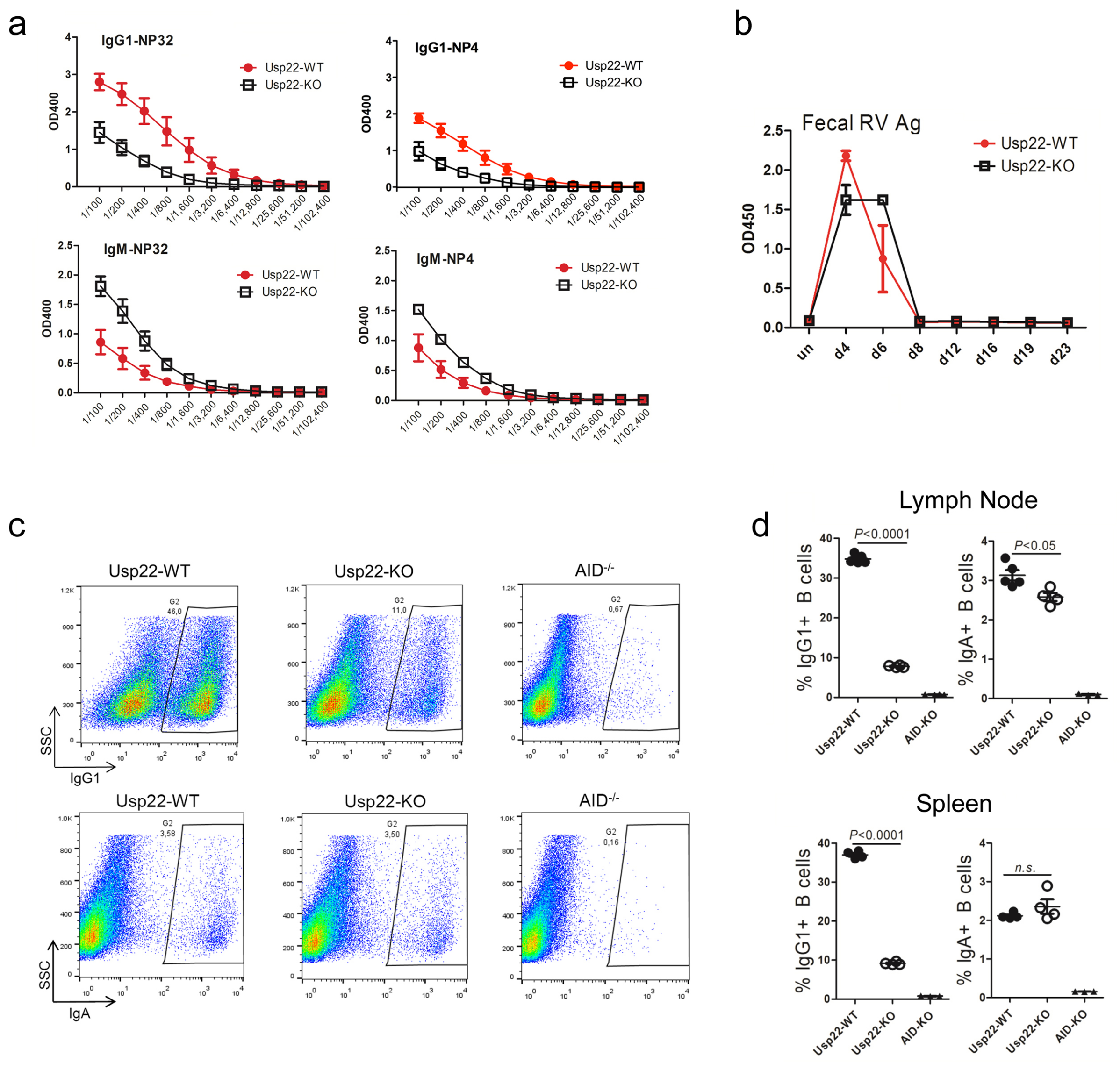

Supplementary Figure 2. CD19-cre-Usp22 KO mice exhibited the defect in IgG but not IgA response. (a) NP25-CGG plus alum were intraperitoneally injected into CD19-cre Usp22 KO or WT littermate mice. At the d22 post-immunization, mice were subjected to NP-specific ELISA to monitor anti-NP IgM or IgG1 response in sera. Data represent three independent experiments each with three to four mice per group. (b) Rotavirus was orally administered to CD19-cre-Usp22 KO or WT littermates. Fecal samples were collected at the indicated time-points post-RV infection, followed by fecal supernatant preparation. The 1:2 dilution of fecal supernatant was used for fecal RV Ag ELISA. Data represent two independent experiments each with three mice per group. (c) Representative FACS plot of ex vivo IgG1 (top panel) or IgA (bottom panel) CSR of spleen B cells isolated from Usp22-WT, CD19-cre-Usp22 KO, or AID-/- mice. IgG1 CSR was induced by LPS plus IL-4 for 4 days. (d) Lymph node or spleen B cells from CD19-cre-Usp22 KO or WT littermates were ex vivo induced to switch to IgG1, and IgA (n= 3-5 samples per group). Data represent two independent experiments and were analyzed using two-tailed unpaired Student t test.

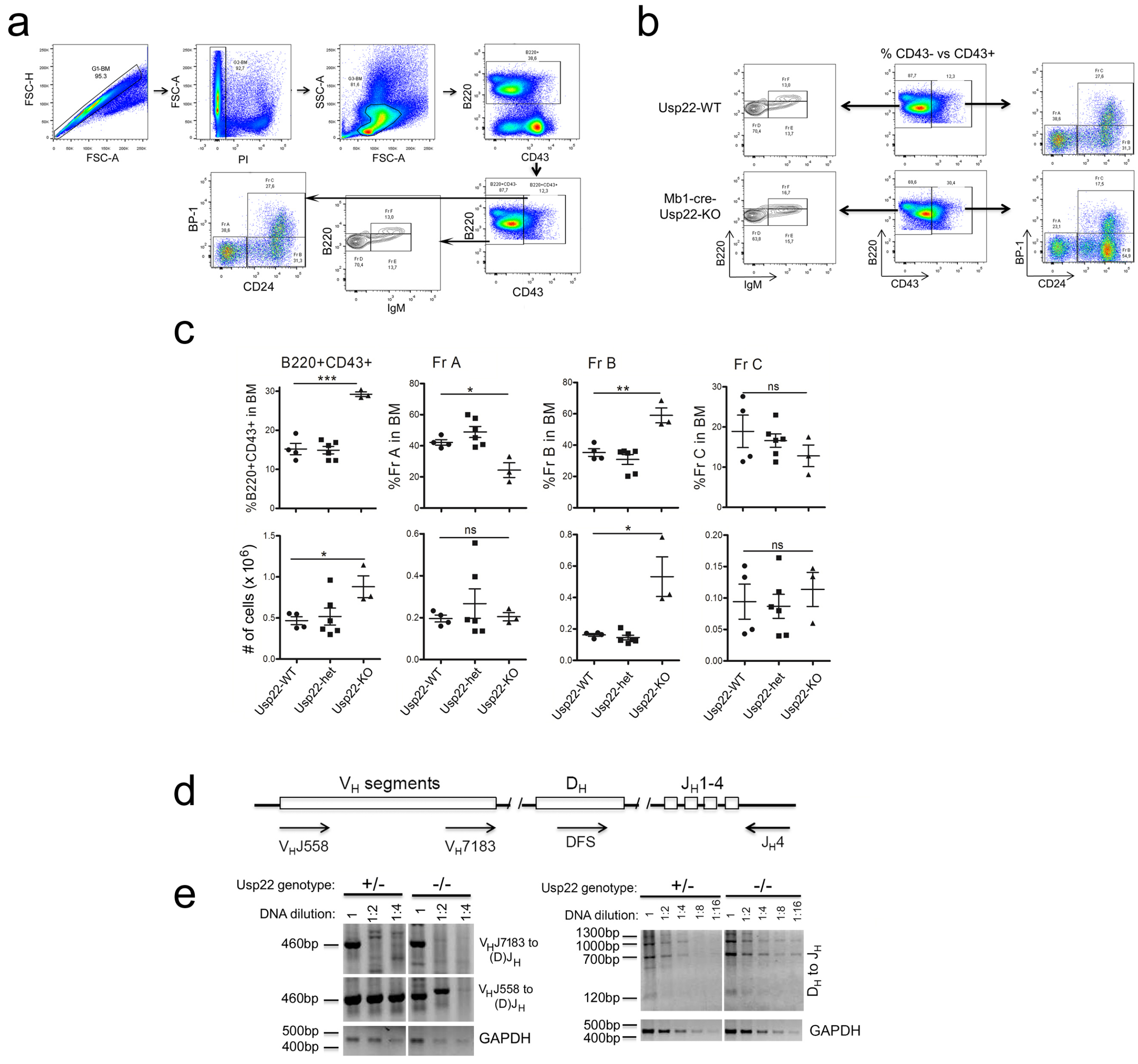

Supplementary Figure 3. Defective pro-B cell development and distal V-(D)J recombination in Mb1-cre-Usp22 KO mice. (a) Gating strategy of the B cell profiling in the BM. (b) Representative FACS plots of B cells in BM of Usp22 WT and Mb1-cre-Usp22 KO mice. (c) The relative percentage (top panel) or absolute number (bottom panel) analysis of B cells of indicated subsets in the BM from Mb1-cre-Usp22 KO and WT littermates (n= 3-6 mice per group). Data were analyzed using two-tailed unpaired Student t test. (d) Schematic of the IgH locus and locations of primers used for distal and proximal V-(D)J, and D-J recombination PCR. (e) Distal VHJ558 and proximal VH7183 to (D)JH segment rearrangement, and DH to JH rearrangement, were assayed in BM pro-B cells of Mb1-cre-Usp22 KO and control WT mice, respectively. GAPDH serves as a quantitative loading control. Data represent two independent experiments.

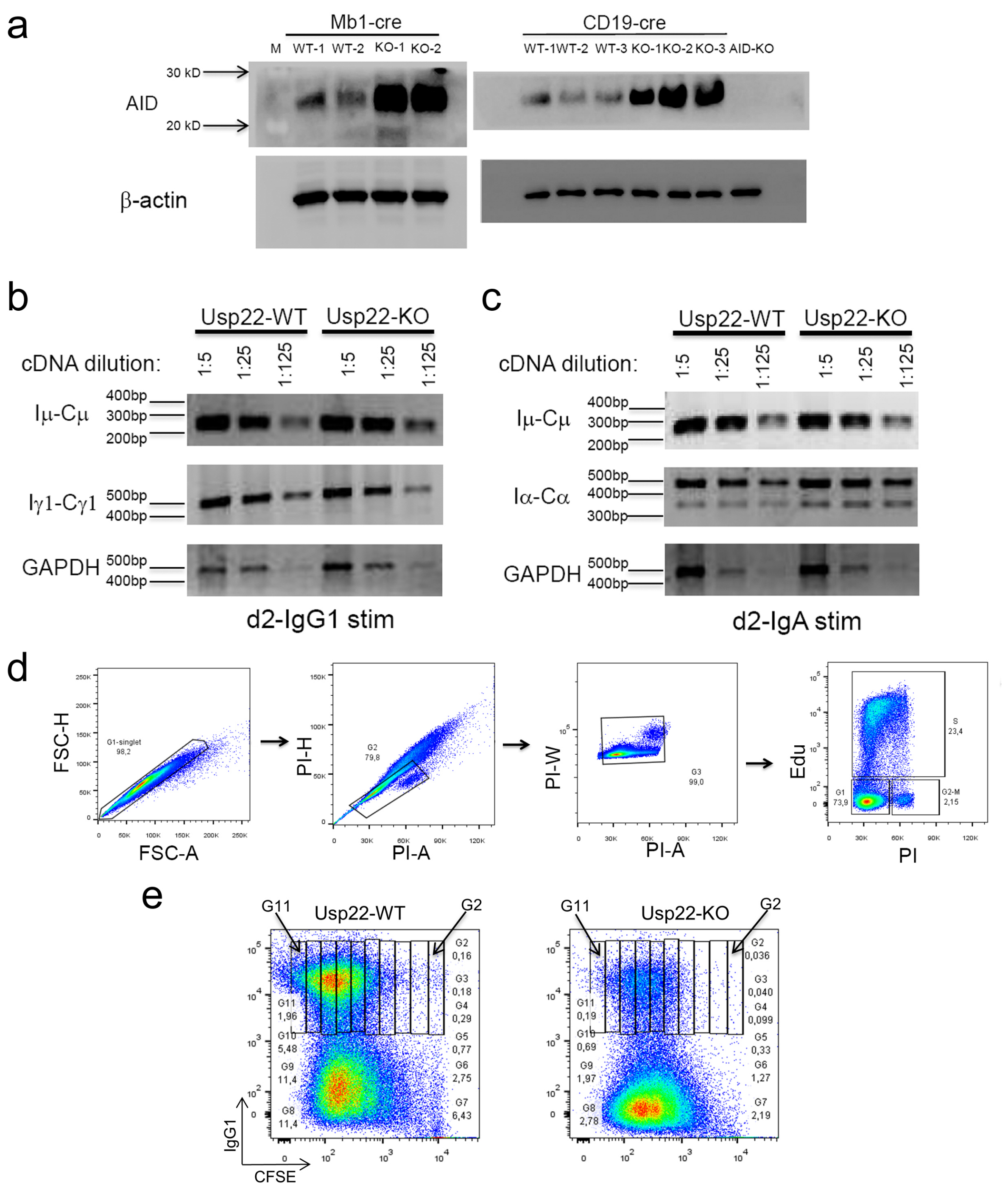

Supplementary Figure 4. Germline transcripts PCR and the ex vivo CFSE-pulsed IgG1 CSR. (a) Uncropped western blot images for the right panel of Figure 3a. (b-c) Splenic B cells from CD19-cre Usp22 KO or WT littermate mice were induced for IgG1 (b) or IgA CSR (c) ex vivo for 2 days. Serially diluted cDNA was used for semi-quantitative PCR to amplify Iμ-Cμ , Iγ1-Cγ1, and Iα-Cα germline transcripts, and GAPDH was used as the internal control. (d) Gating strategy for cell cycle analysis by FACS. (e) Representative FACS plots of CFSE-pulsed spleen B cells of Usp22-WT or CD19-cre-Usp22 KO that were induced to IgG1 CSR by LPS plus IL-4 for 4 days. The gating (G2 to G11) is based on the intensity of CFSE. Data in (b-e) represent two independent experiments.

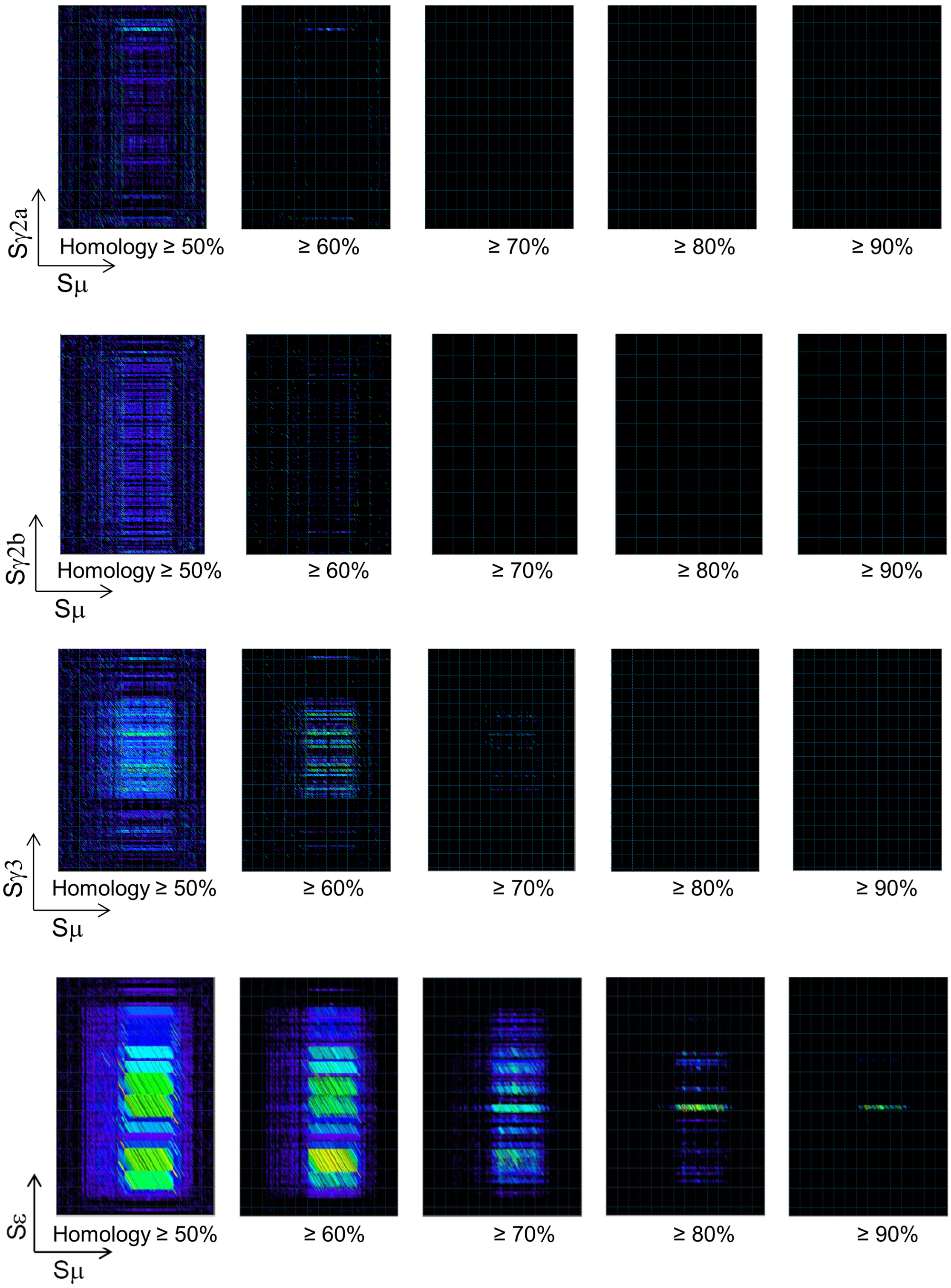

Supplementary Figure 5. Homology analysis of switch region sequences of the IgH locus of C57BL/6 mice. Dot Matrix analysis of the mouse switch regions. The switch region sequence of Sμ were compared to that of Sγ2a, Sγ2b, Sγ3 or Sε in C57BL/6 mice.

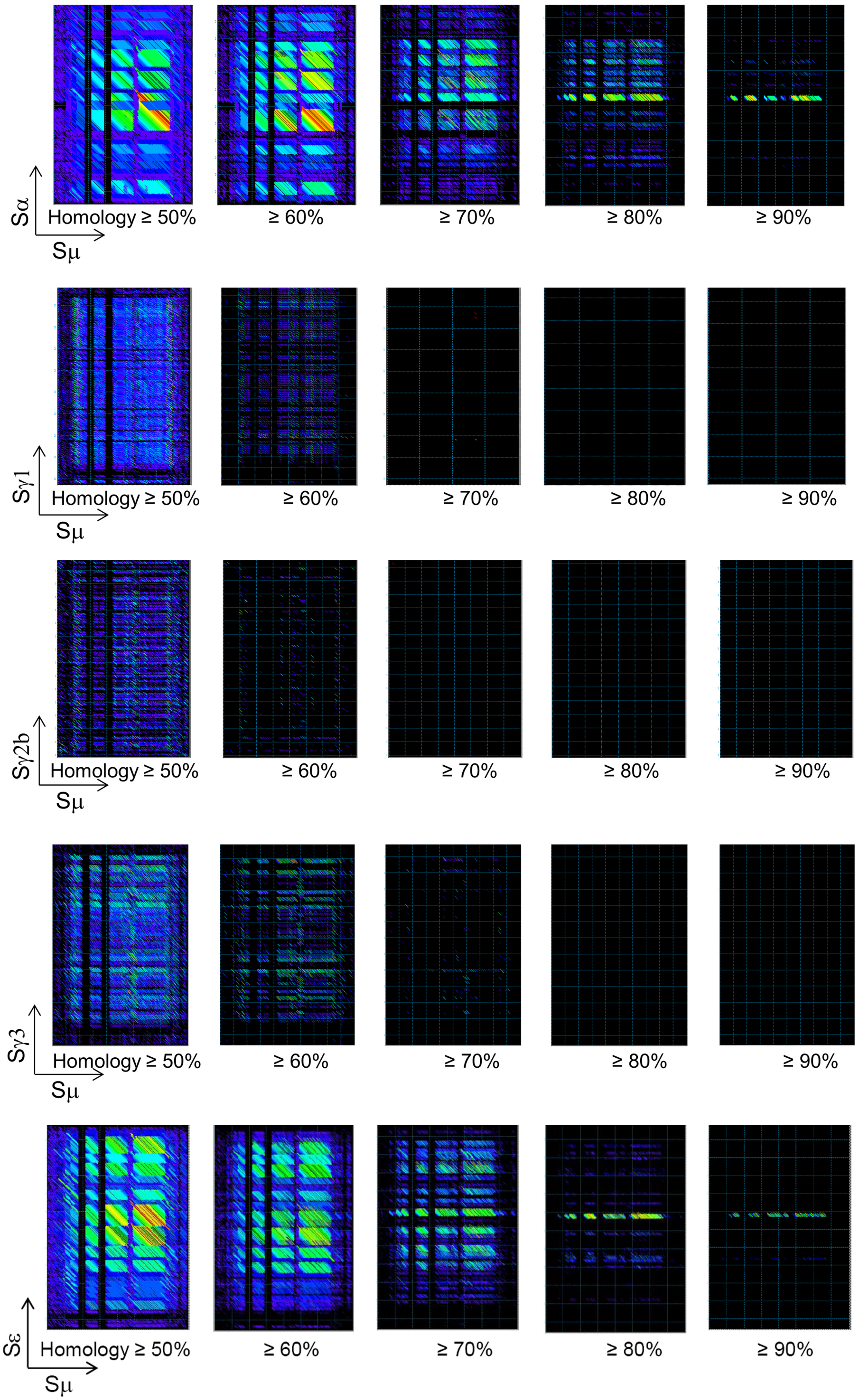

Supplementary Figure 6. Homology analysis of switch region sequences of the IgH locus of BALB/c mice. Dot Matrix analysis of the mouse switch regions. The switch region sequence of Sμ were compared to that of Sα, Sγ1, Sγ2b, Sγ3 or Sε in BALB/c mice.

Supplementary Table 1. Primers used in this study.

Primer	Purpose	Forward Sequence	Reverse Sequence
Usp22	qPCR	5’gctccccacacattccataca3’	5’ggctttcttcccattgtcatca3’
AID	qPCR	5’gcggacatttttgaaatggta3’	5’ttggcctaagactttgaggg3’
HPRT	qPCR	5’cccagcgtcgtgattagc3’	5’ggaataaacacttttttccaaat3’
V_HJ558	V(D)J PCR	5’gcgaagctta(ag)gcctggg(ag)ctt cagtgaag3’	
V_H7183	V(D)J PCR	5’gcgaagcttgtggagtctgggggagg ctta3’	
J_H4E	V(D)J PCR		5’aggctctgagatccctagacag3’
J_H4A	V(D)J PCR		5’gggtctagactctcagccggctccctc aggg3’
DFS	D-J PCR	5’agggatccttgtgaagggatctactact gtg3’	
J_H4C	D-J PCR		5’aaagacctgcagaggccattcttacc3 ’
GAPDH	V(D)J and D-J PCR	5’tccaccaccgtgttgctgtag3’	5’gaccacagtccatgccatcact3’
mS'g1 HS	S’γ1 AID hotspot sequencing	5’gggtgtataaggtaccaggctg3’	5’cttgttactctaccagcctcagc3’
mS'μ HS	S’μ AID hotspot sequencing	5’aatggatacctcagtggtttttaatgg3’	5’gcggcccggctcattccagttcattaca g3’
